# Moral Decision Making: From Bentham to Veil of Ignorance via Perspective Taking Accessibility

**DOI:** 10.3390/bs11050066

**Published:** 2021-05-01

**Authors:** Rose Martin, Petko Kusev, Joseph Teal, Victoria Baranova, Bruce Rigal

**Affiliations:** 1 Department of People and Organisations, Surrey Business School, University of Surrey, Guildford GU2 7XH, UK; r.k.martin@surrey.ac.uk; 2 Behavioural Research Centre, Huddersfield Business School, The University of Huddersfield, Huddersfield HD1 3DH, UK; joseph.teal@hud.ac.uk; 3 Department of Psychology, Lomonosov Moscow State University, 125009 Moscow, Russia; bva06@mail.ru; 4 Institute of Business, Law and Society, St Mary’s University, London TW1 4SX, UK; bruce.rigal@stmarys.ac.uk

**Keywords:** morality, utilitarianism, veil of ignorance, perspective-taking, accessibility

## Abstract

Making morally sensitive decisions and evaluations pervade many human everyday activities. Philosophers, economists, psychologists and behavioural scientists researching such decision-making typically explore the principles, processes and predictors that constitute human moral decision-making. Crucially, very little research has explored the theoretical and methodological development (supported by empirical evidence) of utilitarian theories of moral decision-making. Accordingly, in this critical review article, we invite the reader on a moral journey from Jeremy Bentham’s utilitarianism to the veil of ignorance reasoning, via a recent theoretical proposal emphasising utilitarian moral behaviour—perspective-taking accessibility (PT accessibility). PT accessibility research revealed that providing participants with access to all situational perspectives in moral scenarios, eliminates (previously reported in the literature) inconsistency between their moral judgements and choices. Moreover, in contrast to any previous theoretical and methodological accounts, moral scenarios/tasks with full PT accessibility provide the participants with unbiased even odds (neither risk averse nor risk seeking) and impartiality. We conclude that the proposed by Martin et al. PT Accessibility (a new type of veil of ignorance with even odds that do not trigger self-interest, risk related preferences or decision biases) is necessary in order to measure humans’ prosocial utilitarian behaviour and promote its societal benefits.

## 1. Moral Philosophy 

### 1.1. Bentham’s Utilitarianism 

In the opening sentences of the Introduction to the Principles of Morals and Legislation, Jeremy Bentham describes two sensations, pleasure and pain, and points to their central role in guiding human moral behaviour [1]. Although he was not the first philosopher to establish the importance of these two sensations in prescribing moral conduct (such ideas date back to Epicurus see [2] and Francis Hutcheson [3]), Bentham is frequently credited for it. Much like other enlightenment thinkers, Bentham argued that rather than relying on beliefs or intuition to determine the moral appropriateness of an action, one should instead employ reason. Specifically, Bentham proposed that a morally permissible action is one that produces the greatest happiness of the greatest number; in that, reducing pain and increasing the pleasure of those affected. Therefore, Bentham [1] intended pleasure to be maximised and pain to be minimised where possible, and accordingly suggested that pleasure can be measured in terms of *utility*, and pain measured in terms of *disutility*. Bentham coined this moral doctrine The Greatest Happiness Principle (hereafter, Utilitarianism).

Much like other consequentialist theories, utilitarianism encompasses the logic of utility maximisation: the ends (if, the best outcome) justify the means. In other words, as long as the end goal maximises utility, then any potentially egregious act required in order to deliver the goal can be justified. However, there is an important distinction between utilitarianism and other moral consequentialist theories. For example, egoistic consequentialism is characterised by the maximisation of utility for self-interest, in the absence of the interest of others [4]. Alternatively, altruistic consequentialism aims to maximise utility for others, in the absence of self-interest [4,5]. Conversely, the goal of utilitarian consequentialism is to maximise utility for the greatest number of people, or according to Bentham’s slogan: “it is the greatest happiness of the greatest number that is the measure of right and wrong” [6] (p. 3). 

Since Bentham’s utilitarianism was originally a proposal for legislative purposes, he proposed that those people with the authority to make a decision should behave as impartial spectators (i.e., observant bystanders). This differs once again from egoistic and altruistic consequentialism, since egoistic decisions are intended to directly benefit the decision-maker, and altruistic decisions can in some cases cause the decision-maker harm [7]. Utilitarianism is therefore a unique consequentialist theory in two ways: (i) the aim is to maximise utility for the greatest number of people, and (ii) when employing utilitarianism in its purist form, the decision-maker should be impartial. 

Bentham’s formation of utilitarianism was focused on pleasures and pains in terms of their *quantity*. However, given that pleasures and pains are not easily quantifiable, Bentham suggested a method (known as the felicific calculus) to measure them based on 7 dimensions. The 7 dimensions included a pleasure or pain intensity, duration, certainty/uncertainty, proximity/remoteness, fecundity (the probability that it will be followed by a sensation of the same kind), purity (the probability that it will not be followed by a sensation of the opposite kind), and extent (the number of people the sensation extends to). Moreover, John Stuart Mill [8], extended Bentham’s proposal to incorporate *quality of pleasure*. Specifically, Mill [8] proposed that: (i) pleasures can differ from one another based on qualitative distinctions, (ii) some pleasures are considered higher than other pleasures based on these qualitative distinctions and (iii) the qualitative distinction between pleasures concern whether pleasures require human or limited animal faculties in order to be experienced [9]. Mill [8] believed that mental pleasures are superior to bodily pleasures since both can be experienced by humans, whereas only the latter can be experienced by animals. Mill therefore argued that higher pleasures were more worthwhile pursuing, particularly if a person is faced with a choice between experiencing the sensation of a lower or higher pleasure.

### 1.2. Kantian Deontology

Another way to position utilitarian ethics, is to establish how it contrasts from other ethical theories. One major ethical theory incompatible with utilitarianism is deontology. Much like utilitarianism, deontologists such as Immanuel Kant [10] believe that moral appropriateness can be determined by reason. However, unlike utilitarian thinkers’ whose notion of reason is based on whether an act will result in particular pleasures or pains, deontologists believe that behavioural acts themselves determine morality. Therefore, deontologists are concerned with behavioural acts of humans and whether such acts are consistent with moral rules.

For example, deontologists such as Kant would argue that sacrificing someone’s life is an impermissible act that no one under any circumstance should engage in. Utilitarianists may also agree but only if refraining from sacrificing someone’s life will result in the greatest happiness for the greatest number. In some circumstances this is not the case. For example, consider the following scenario (the trolley dilemma) originally assembled by Foot [11] (but adapted by Greene et al. [12]):

A runaway trolley is headed for five people who will be killed if it proceeds on its present course. The only way to save them is to hit a switch that will turn the trolley onto an alternate set of tracks where it will kill one person instead of five. Ought you to turn the trolley in order to save five people at the expense of one? (p. 2105).

In response to this scenario and question, a deontologist would not in any case permit the turning of the trolley as this act would involve sacrificing someone’s life. Consequently, 5 people will die, and 1 will live. Alternatively, a utilitarian would permit the turning of the trolley since this act of sacrificing one person will result in saving the lives of the greatest number of people. Therefore, 1 person will die and 5 will live. However, it is important to note that simple utility ratios (choosing to save 5 people over 1 person) may be overruled by other utility maximisation goals. For example, if it is known (available in the context) that the 1 man on the track is an accomplished biochemist on the verge of discovering the cure for a deadly disease (whose discovery will go onto save millions of people) then a utilitarian should opt to save the 1 biochemist over the 5 non-biochemists who will not go on to bring about such utilitarian consequences. Moreover, this example demonstrates another important distinction between utilitarianism and deontology; whilst a deontologist would not change their choice in light of the contextual information (murder is impermissible in all situations), a utilitarian decision maker would always maximise the utility given the available information. This is also the main theoretical premise of the normative decision-making theory (see Section 2).

## 2. Normative Decision-Making

Bentham’s utilitarianism has had a lasting impact on behavioural science and economics, including the approach to theorising about- and predicting human behaviour. Accordingly, normative utilitarian decision-making theorists (e.g., [13,14]) expect agents to maximise utility and/or minimise disutility (i.e., maximise gain and/or minimise loss) when making economic decisions (see [15,16]). In particular, and not too dissimilar to Bentham’s felicific calculus, normative theories assume that human agents make utility calculations based on objective known values (such as money and probability) and choose the option that maximises utility (or, in the context of inevitable loss, the option that minimises disutility). However, results from behavioural science and psychology research reveal issues with (and violations of) normative utilitarian principles and assumptions. For example, the psychology of decision making in the domains of gain and loss is different, the behaviours in these two domains are largely dissociated and dependent on experience [17,18,19,20,21,22,23]. These findings from behavioural science and economics are fundamental in informing research in moral decision-making, since many hypothetical moral problems have been constructed in order to test normative utilitarian assumptions.

### 2.1. Rationalist and Intuitionist Approaches to Moral Decision-Making

In the field of moral decision making, there are two main approaches to understanding how humans process moral problems: (i) moral rationalists who believe that controlled moral reasoning guides human moral choices, and (ii) the moral intuitionists who argue that human moral choices are the result of automatic intuitive responses. According to the rationalist approaches to moral decision-making, controlled cognitive processing is a requirement when making moral decisions (e.g., [24,25,26,27]). Kohlberg’s [28,29,30] theory of moral development emphasises this rationalist approach. Accordingly, Kohlberg’s paradigm was based on children’s responses to hypothetical moral dilemmas, where children indicated whether a target behaviour is morally right or wrong and justified their reasoning (see for dilemma examples, [31]). Based on his experimental findings, Kohlberg [28] suggested that children advance their moral reasoning capabilities through 3 developmental levels: (i) the pre-conventional, (ii) the conventional and (iii) the post-conventional. The pre-conventional level is characterised by punishment avoidance and egoistic self-interest. At this level, an individual can establish right and wrong based external punishments as opposed to an internal experience of guilt. Moreover, the individual behaves according to their own self-interest with little or no concern for others. The conventional level is defined by the ability of the individual to conform to social norms and adopt an internalised rule-driven understanding of moral conduct. Finally, at the post-conventional level, the individual develops their own moral principles and opinions, which may deviate from law. These principles could be based on philosophical viewpoints, context, engage perspective-taking (see [32]) and change on a case-by-case basis. Therefore, Kohlberg’s theory of moral development suggests that we approach moral problems using reasoning; an ability that gradually matures over the course of development.

In opposition to rationalist models of moral decision-making, moral intuitionists claim that human assessments of moral permissibility result from fast and emotionally driven intuition (e.g., [33,34,35]). In particular, Haidt [33] argues that decision-makers initially react to moral problems with an intuitive response and attempt to employ reason only after a judgement has been made (as a post-decisional justification). Haidt [33] points to the example of a hypothetical situation where a family’s dog dies after being run over by a car in front of the family home. The family consequently decide to make use of the dead dog by cooking its meat and eating it. In response to this and other similar scenarios participants often make an initial judgement such as “this behaviour is wrong”. However, when asked to elaborate further, participants attempt to justify their choice, employing reasoning after their intuitive reaction. In some cases, this results in *moral dumbfounding*, where participants claim that a behaviour is “just wrong” despite not entirely knowing why or being able to rationally justify it.

Such intuitive responses could be the result of innate evolutionary mechanisms (e.g., [36,37]), however some theoretical and empirical work suggest roots in reinforcement learning (e.g., [38,39,40,41,42]). For example, Skinner [38] provides a different account of moral development to that of Kohlberg [28], contending that individuals learn moral rules through simple reinforcement learning processes, where learned rules become intuitive reactions/aversions to particular outcomes. Therefore, based on reinforcement histories, individuals learn to associate particular behaviours with pleasure or pain related outcomes. According to fairly recent experimental findings [40], successful reinforcers that cause deviations from utilitarian choice can be as simple as verbal cues indicating whether a behaviour is correct or incorrect. As a result of learning moral rules though a reinforcement learning task, participants’ moral choices resembled intuitive rule-governed responses as opposed to utilitarian maximisation strategies that would reflect controlled reasoning.

### 2.2. The Dual Process Theory of Moral Decision-Making

Both rationalist and intuitionist approaches to moral decision-making provide convincing arguments for their respective schools of thought. However, Greene et al. [12,43,44] proposed that decision-makers employ either controlled processing *or* emotional intuition when tasked with making moral choices. Greene et al.’s [12] initial idea was based on a philosophical puzzle regarding how humans respond inconsistently to different variations of the same hypothetical moral dilemmas (see [11,45]). For example, consider once again the trolley dilemma but this time paired with the footbridge dilemma [12]:

The Trolley Dilemma:

A runaway trolley is headed for five people who will be killed if it proceeds on its present course. The only way to save them is to hit a switch that will turn the trolley onto an alternate set of tracks where it will kill one person instead of five. Ought you to turn the trolley in order to save five people at the expense of one?

(p. 2105)

The Footbridge Dilemma:

You are standing next to a large stranger on a footbridge that spans the tracks, in between the oncoming trolley and the five people. In this scenario, the only way to save the five people is to push this stranger off the bridge, onto the tracks below. He will die if you do this, but his body will stop the trolley from reaching the others. Ought you to save the five others by pushing this stranger to his death?

(p. 2105)

Greene et al. [12] noted that consistent with utilitarian moral principles, most people will agree that killing the 1 person in order to save 5 in the trolley dilemma is permissible (resembling controlled processing involving simple utility calculations). However, in the footbridge dilemma the majority of people often refrain from pushing the 1 man onto the tracks in order to save 5 (resembling harm-averse emotional intuitions). This inconsistency creates a puzzle for normative theorists since different representations of the identical (in terms of utility options/outcomes) choice problems result in different preferences (utilitarian or non-utilitarian). Some authors contend that these dilemmas elicit different moral choices because each dilemma differs in the level of personal involvement. For example, the footbridge dilemma is considered personal because it requires the decision-maker to actively push a man to his death, whereas the trolley dilemma involves a mechanism that distances the decision-maker’s actions from the harm outcome [11,45]. Greene et al. [12] further hypothesised that personal and impersonal dilemmas elicit different psychological processes.

Suitably, Greene et al. [12] investigated this hypothesis by employing fMRI technology to map activity of emotional and working memory related regions of the brain whilst participants read and answered personal and impersonal moral dilemmas. The authors found that decision-makers were indeed more utilitarian in their responses to impersonal dilemmas compared to their responses to personal dilemmas. Moreover, the neuroimaging findings revealed that areas of the cerebral cortex associated with emotion (Brodmann’s Area 9, 10, 31 and 39) showed heightened activity when participants read personal moral dilemmas (e.g., the footbridge dilemma) compared to when they read impersonal moral dilemmas (e.g., the trolley dilemma). Moreover, areas of the cerebral cortex associated with working memory (Brodmann’s Area 7, 40 and 46) showed heightened activity when participants read about impersonal moral dilemmas competed to when they read personal moral dilemmas. The authors therefore likened their findings to the dual process theory of decision-making [46]; where one of 2 possible systems can be employed to process information, including system 1 (fast and intuitive, emotionally driven processing), and system 2 (slow and controlled cognitive processing). Accordingly, when considering an impersonal moral dilemma, system 2 processing is employed, which calculates the utility maximising option (utilitarian choice). However, when considering personal moral dilemmas, system 1 processing competes with and dominates system 2 processing which results in emotionally driven responses (non-utilitarian choice) that avert choices that actively cause harm [12].

Interestingly, the time taken to make utilitarian decisions also differed between dilemma types. For example, it took longer for people to make utilitarian decisions in response to personal dilemmas than it did to make utilitarian decisions in response to impersonal dilemmas. This indicates an interruption in the processing of personal dilemmas which is most likely the result of emotional activations identified in the fMRI data [12].

Taken together, these findings indicate that utilitarian judgements are the result of slow and controlled processing whereas deontological judgements are the results of fast and intuitive emotional processing. The dual process theory of moral decision-making therefore provides evidence for both rationalist and intuitionist accounts of moral decision-making: moral decisions can be the result of either controlled cognitive or emotional intuitive processing and this is dependent on characteristics of the moral problem (e.g., whether the moral scenario requires personal or impersonal involvement; [12,43]). However, this theoretical proposal was recently challenged ([47,48,49]; Section 2.3).

### 2.3. Contextual Accessibility in Moral Decision-Making Tasks

Many cognitive psychology and decision-making theorists have focused their efforts on investigating how the human mind processes information. In particular, some decision-making researchers argue that our choices are the result of the methods used to process the choice options (e.g., [12,20,46,50]). For example, dual-process theorists of moral behaviour claim that information can be processed via two competing systems, with the employment of each system resulting in different choices and judgements. Moreover, according to Unconscious Thought Theory human choice preferences also depend on whether we have processed decision-making information at a conscious or unconscious level [50]. However, whilst the processing of information is important to the formation of judgements and decisions, the way that information is processed in the first place is highly dependent on the construction of the information itself (e.g., [19,20,47,51,52,53]). For instance, how descriptive information is presented to participants can greatly influence the individual’s choice behaviour. Tversky and Kahneman [52] demonstrated that the framing of decision problems influences people’s risk preferences. In particular, decision-makers were found to make risk-averse choices when they read scenarios framed in terms of gain, and risk-seeking choices when they read scenarios framed in terms of loss. The framing effect offers an interesting example of how the formulation of information can impact risky decisions.

More recently however, Kusev et al. [47] have explored the influence of enhanced accessibility to information on moral judgements. In their proposal, Kusev et al. [47] argued that traditional moral dilemmas based on Thomson’s [45] trolley paradigm offer participants limited accessibility to dilemmas information and task, rendering the scenarios cognitively challenging. In their version of the trolley and footbridge dilemmas, Kusev et al. [47] have found that enhanced accessibility to dilemma information and task (when people are presented with the full implications of their actions), they are more likely to weigh their choices in a manner that is consistent with utilitarian ethics. Importantly, Kusev and colleagues also found that people took the least time to make their decision when they were given the full information and when they chose to save 5 lives at the expense of 1, irrespective of whether the involvement was personal or impersonal. Therefore, enhanced accessibility of utilitarian outcomes through comprehensive information about moral actions and consequences boosted utility maximisation in moral choices, with rational choices taking less time.

## 3. Moral Perspective-Taking Accessibility

### 3.1. Autonomous Vehicles: A Very Real Moral Problem

So far, in the moral decision-making research literature, hypothetical moral dilemmas have been implemented as tools to understand human cognition. However, as argued in an extensive review by Bauman et al. [54], hypothetical moral dilemmas often detail considerably unlikely events; the scenario itself therefore lacks mundane realism (how likely the scenario would occur in decision-makers daily life). However, recent advances in the development of artificially intelligent machines (e.g., autonomous vehicles) have resulted in trolley-like scenarios becoming a very real moral problem. For example, much like the decision the participant is expected to make in response to the trolley dilemma, autonomous vehicles can be pre-programmed to choose whose life to save in crash scenarios (and take into account all possible situational factors). Accordingly, AVs will be programmed to make passenger-protective decisions (protecting the passenger at all costs), or to make decisions compatible with utilitarian ethical principles (protecting the greatest number of people); see later in the section further discussion. Moreover, this is particularly important as the AVs (as well as the passengers and pedestrians), will always be involved in inevitable moral dilemmas, decisions, and collisions. This is because of the unpredictability of other human drivers, cyclists, and pedestrians’ behaviour (as well as animals and debris). Accordingly, the pre-programmed moral decision (utilitarian pro-social or passenger protective) must be made by a human (e.g., the car manufacturer), approved by the legislator/policy maker, and endorsed by human customers (buying the prosocial or passenger protective AV). This has created a new application of moral decision-making research, and accordingly new and realistic moral problems that must be addressed before autonomous vehicles become available to the public [55,56]. Therefore, in this article, autonomous vehicles will be discussed along with the inevitable moral dilemmas that policy makers and car manufacturers face when implementing ethical algorithms.

Autonomous Vehicles (AVs) are cars that can take control of some or all aspects of driving including acceleration, deceleration, steering, and monitoring of the driving environment [57]. Importantly, this means that AVs can replace human drivers in many or all of the highly demanding tasks required to operate a vehicle. Although, AVs may sound like science fiction, early conceptions and models have existed since the 1920’s when the first radio-controlled driverless car was tested by the US military. While radio-controlled cars were not technically autonomous, they were constructed and tested with the intention of relieving drivers of driving tasks and promoting driving safety [58]. Since the 1930’s the fictional concept of actual AVs that drive themselves and learn complex road networks have appeared in numerous sci-fi novels and films (see [58]). AVs are therefore not a contemporary idea.

Whilst all AVs occupy autonomous driving features, not all AVs possess the same level of autonomy. The Society of Automotive Engineers (SAE) have defined 6 levels of automobile automation ranging from no automation (non-autonomous; level 0) which applies to cars without system operated driving assistance features (e.g., lane discipline) to full automation (level 5), where the AV occupies full autonomy over acceleration, deceleration, steering and monitoring of the driving environment in the complete absence of interference from human passengers (see [57] for all AV levels). Importantly, for the purpose of this article, the definition of AVs will be restricted to fully automated level 5 AVs.

AVs have received widespread multidisciplinary attention from researchers in artificial intelligence, engineering, transport, law, philosophy, psychology and business (e.g., [48,59,60,61,62,63,64]. Moreover, many car manufacturers such as Ford, Mercedes and Tesla, as well as technology companies including Google and Uber are currently involved in developing and testing autonomous driving technology [65]. The introduction of such vehicles presents many benefits, the most obvious being that AVs will relieve current drivers from highly demanding driving-related tasks. However, AVs can also be utilised by non-drivers too since they will be capable of transporting disabled people, the elderly and children [66]. Moreover, AVs are also predicted to significantly reduce road traffic. According to Bose and Iannou [67], only 10% of cars on a single highway segment need to be autonomous for there to be a significant improvement in highway congestion. Furthermore, despite the introduction of AVs potentially increasing the number of people on UK roads, AVs are also predicted to emit less greenhouse gasses into the atmosphere than non-autonomous cars due to the reduction in energy-wasting human driving errors and inefficiencies [68].

Perhaps one of the most important implications of replacing human drivers with automated transport systems is the anticipated reduction in the number of road accidents often caused by human factors such as drink driving, fatigue, and human error [61,69]. Accordingly, Fagnant and Kockelman [61] estimate that AVs will (at the very least) prevent 40% of road accidents. Nevertheless, given the unpredictability of other human drivers, cyclists, and pedestrians’ behaviour (as well as animals and debris), it is inevitable that AVs will still be involved in collisions. However, AVs can be pre-programmed to scan the environment and calculate the most moral course of action within seconds. Yet, what constitutes the most moral course of action must first be defined by humans.

Pre-programming AVs comes with many ethical, legal and safety implications (cf. [63]). One concern relates to the possible ethical principles that will be embedded into AV algorithms. For instance, in preparation for potential unavoidable collisions, AVs could be programmed to make passenger-protective decisions (protecting the passenger at all costs), or to make decisions compatible with utilitarian ethical principles (protecting the greatest number of people). Of course, as pointed out by [64], AVs will never actually make a moral decision; instead, AVs will follow through pre-determined decisions that have been configured by humans. This therefore leads to the issue of who gets to decide how AVs should be programmed and how this may affect the AVs utilitarian or nonutilitarian behaviour. For example, the design of ethical algorithms might be informed by policymakers who tend to impose limits on individual freedom in order to benefit the overall community, which suggests that they might opt for utilitarian AVs (see [65]). Accordingly, in everyday driving it is illegal to drive over the designated speed limit. Whilst this restriction may limit the individuals’ driving freedom, having speed regulations in place contributes to the safety of the wider community as a whole. However, policymakers may not be tasked with regulating mandatory ethical standards for AVs. Alternatively, the car manufacturers themselves may have the power to choose how to program their vehicles, and might opt for passenger-protective cars since they may be easier to market to consumers [60]. For example, in a 2016 interview, Mercedes Benz executive Christoph von Hugo assured his customers that in the event of a collision, future Mercedes AVs will prioritise the lives of their passengers [55].

Some authors have entertained the idea that the car buyers themselves should have a say in the ethical behaviour of their own cars [70]. Accordingly, AVs could possess a personal ethics setting (PES), where AV owners can adjust the ethical setting in their car, selecting between protecting themselves or protecting the greatest number of people. However, the proposal of a PES has also been received with criticism, since according to game theory predictions, people will put their own personal safety over the social welfare of the community, ironically resulting in an increased probability that the driver will die in an accident [71].

According to utilitarian theory (e.g., [1]), the most moral course of action would be to programme AVs to minimise overall harm. Normative theorists would also argue that utilitarian AVs are the most rational cars since they maximise utility [12,14]. Likewise, in moral psychology, utilitarian choices are often considered desirable and focal point in the exploration of moral decision-making [12,44,47]. Utilitarian AVs have accordingly been perceived by many authors as the most prosocial vehicle and therefore the most morally appropriate vehicle for public use [56,60,71]. For instance, existing driving laws are utilitarian in their very nature since they limit individuals’ freedom in order to promote the greatest overall safety for the driver and other people. Moreover, one of the major goals of replacing human drivers with AVs is that they are expected to reduce the number of deaths and injury’s caused by human driving errors (thus minimising harm). Therefore, utilitarian AVs fit in with current UK driving regulations and with the general harm minimising goals of autonomous driving technology. However, whilst introducing utilitarian AVs may be the most prosocial and beneficial to societal wellbeing, this does not necessarily mean they will be received well by the public.

### 3.2. Veil of Ignorance: The Moral Perspective-Taking Accessibility Approach

The success of any business is highly dependent on consumers perception of its product. Accordingly, Gogoll and Müller [71] argue that if the ethical standards of AVs do not match the moral preferences of the potential consumers, then marketing AVs will be a challenging feat. Given that it is highly unlikely that AVs will be embedded with a PES, it is important that the consumers moral preferences towards AV ethics are taken into account when AV ethical algorithms are developed. However, empirical research in psychology has revealed that people’s moral preferences towards AVs are not straightforward. For instance, utilitarian moral preferences regarding the ethical programming of AVs have been found to vary as a function of gender, culture, context and religious beliefs [59]. Moreover, utilitarian moral preferences can be swayed by contextual factors such as how many people are involved in a collision and who is at fault [59,62].

**Figure 1:**
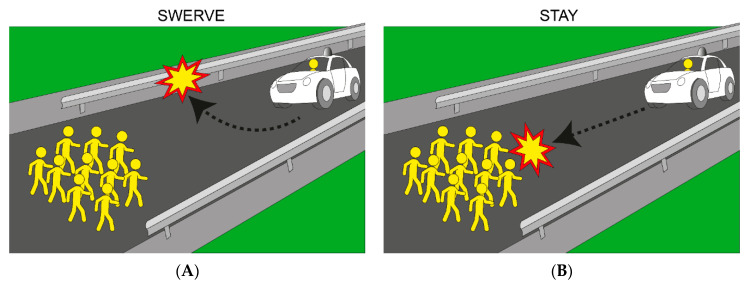
A visual representation of an AV crash scenario. Note. Panel **A**: a utilitarian AV swerving into a barrier, sacrificing the lone passenger in order to save the 10 pedestrians. Panel **B**: a non-utilitarian AV staying on its path, sacrificing the 10 pedestrians in order to save the lone passenger.

Perhaps one of the most intriguing empirical findings (see [60]) reveals utilitarian preference inconsistencies within the same people, where people do not want to own the AV that they perceive to be the most morally appropriate. Accordingly, in study 3 of Bonnefon et al. [60], the experimenters presented participants with a variation of a scenario where they had to imagine themselves inside an AV that is about to crash into a group of 10 pedestrians in the road. The participants were told that the AV could be programmed to swerve off to the side of the road where it will impact a barrier and kill them (sparing the 10 pedestrians) or it could be programmed to stay on its current path and kill the 10 pedestrians (sparing themselves; see Figure 1 for a visual depiction of this scenario). Participants were then asked to rate which AV they perceived to be most moral, as well as to indicate their willingness to buy each AV. Bonnefon et al. [60] found that participants judged utilitarian AVs as the most morally appropriate for societal use, yet they wanted to buy passenger-protective models themselves. Therefore, moral preferences towards AVs depend upon the decision-maker’s role: as citizens, people want prosocial utilitarian AVs but as consumers, people opt for passenger-protective models [56], exemplifying a social dilemma.

**Figure 2:**
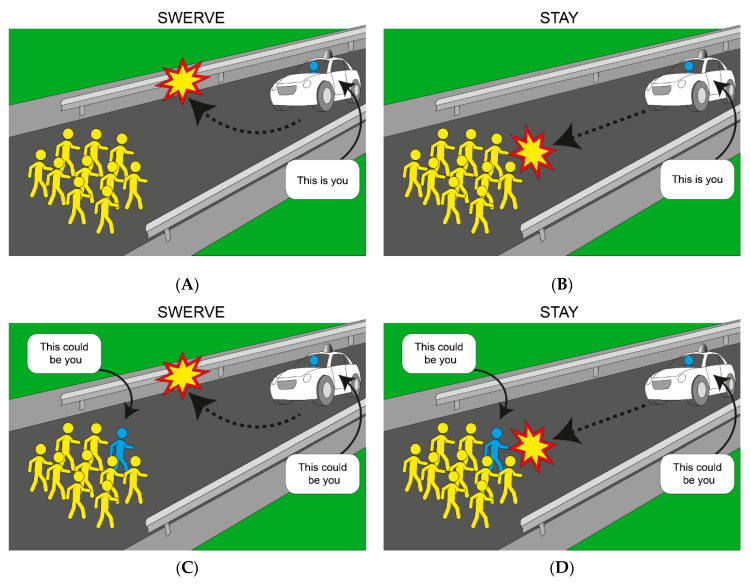
A visual representation of an AV crash scenario with partial and full PT accessibility. Note. Panel **A**: a utilitarian AV with partial PT accessibility. Panel **B**: a non-utilitarian AV with partial PT accessibility. Panel **C**: a utilitarian AV with full PT accessibility. Panel **D**: a non-utilitarian AV with full PT accessibility.

Bonnefon et al.’s [60] findings have important implications for policymakers, car manufacturers and the general public. However, in a commentary article Martin et al. [48] argued that these findings in which such implications are based may simply be an artifact of the experimental materials presented to participants. In particular, in Bonnefon et al.’s [60] AV crash dilemmas, the participants engage in a perspective-taking task where they are required to imagine themselves (or a stranger or even themselves accompanied by a family member) inside an AV. The critical issue here is that participants are not offered the corresponding perspective of a pedestrian. Therefore, whilst participants are presented with a perspective-taking task, the task itself lacks accessibility to the two situational perspectives (i.e., the passenger and one of the pedestrians). Perspective-taking (PT) is the ability to mentally represent how another person is feeling by (i) imaging how another person feels in their situation or (ii) imaging how you would feel in another person’s situation [72,73,74]. When PT is partial (not all situational perspectives are accessible), decision-makers make choices and judgements based on limited information which could lead to decision-making biases [47,48,49]. Accordingly, Martin et al. [49] introduced a new definition and method of PT, full PT accessibility, where participants have access to all situational perspectives in a particular scenario (for example, see Figure 2).

In the context of AV crashes, offering partial PT accessibility to moral dilemmas is problematic, considering that by default AV buyers will not only be passengers of AVs but will be pedestrians too (e.g., as soon as they exit their vehicle). Accordingly, Martin et al. [49] argued and established that AV buyers should be provided with access to both the passenger and pedestrian perspectives (even odds of being a passenger or pedestrian—as a principle of impartiality), or in other words full PT accessibility in order to value, buy and ride the utilitarian AVs. Accordingly, Martin et al. [49] found that presenting participants with AV dilemmas with full PT accessibility eliminated the behavioural inconsistency between participants’ non-utilitarian purchase behaviour and their utilitarian judgements of moral appropriateness. Moreover, the authors also established that moral dilemmas with full PT accessibility boost both participants’ utilitarian purchasing behaviour and utilitarian judgements of moral appropriateness.

The PT accessibility theory by Martin et al. [49] is motivated by a thought experiment described by Rawls [75] where an agent must imagine they have no knowledge of their personal attributes or circumstances (e.g., their gender, race, class or whether or not they have a disability) and are therefore shrouded by a ‘Veil of Ignorance’ (VOI). Adopting VOI reasoning is expected to induce impartiality in ostensibly selfish agents; as VOI is an unbiased state which may accordingly serve useful in organising a fair society. One major assumption of Rawlsian VOI is that agents are motivated by self-interest. For instance, if an agent is tasked with the role of organising society whilst also aware of their personal attributes and circumstances, they are expected to make (biased) decisions that benefit their own profile over others.

More recent interpretations and development of VOI (e.g., [76,77]) principles include empirically induced self-interest and impartiality—as even odds of being each of the people affected by the moral decision. Accordingly, Huang et al. [77] proposed that in VOI reasoning tasks participants may adopt utilitarian behaviour, simply because they are selfish and aim at maximising their own odds of a good outcome. However, Martin et al. [49] proposed and established a more inclusive VOI framework, that takes into account PT accessibility. In order to appreciate Martin and colleagues’ proposal, we will point out some of methodological constrains of Huang et al.’s VOI. For example, Huang’s VOI reasoning tasks are always ‘personal’ (participant involvement), demanding and inducing selfishness (“Please respond from a purely self-interested perspective”), and do not provide the decision makers with the opportunity to engage in full perspective taking. Moreover, as pointed out by Martin et al. [49], Huang and colleagues employed even odds of being each of the people affected by the moral decision, making the moral decision a matter of ‘risk’, rather than ‘ambiguity’. For example, “If the law requires the autonomous vehicle to swerve in such a situation, you have a 1 out of 10 chance of dying and a 9 out of 10 chance of living. If the law forces the autonomous vehicle to stay on its current path, you have a 1 out of 10 chance of living and a 9 out of 10 chance of dying.” (see supplemental materials, [77]); the swerve option is risk-averse (and happened to be utilitarian), and the stay option is risk-seeking (and happened to be non-utilitarian), and therefore rendering the odds uneven (see [49]). In addition to creating a risky version of VOI, Huang et al. [77] also induce selfishness in participants (“Please respond from a purely self-interested perspective”) which may have further fuelled risk-averse precautionary preferences, which in this task also happened be the utilitarian.

In order to combat these limitations and confounds, Martin et al. [49] proposed and employed a new type of VOI with full PT accessibility—even odds of being a pedestrian or passenger in crash scenarios (eliminating the opportunity of making self-interest decisions—e.g., by selecting the ‘risk averse’ option, which happened to be utilitarian), and thus impartiality. This is in a sharp contrast to Huang and colleagues’ proposal of even odds of being each of the people in the moral scenario.

Furthermore, in Martin and colleagues’ PT accessibility tasks, participants were not primed to act selfishly and there was no incentive for them to do so, given the riskless design of the decision-making task. Martin et al.’s [49] findings reveal that with full PT accessibility (participants are made aware that they could be either a passenger or pedestrian; even odds of being a passenger or pedestrian, and therefore with even 50/50 chance to die/live as passenger or pedestrian), participants are more utilitarian in their moral preferences, purchasing values and are more willing to buy and ride utilitarian AVs. Accordingly, Martin et al. [49] proposed that full PT accessibility does not trigger self-interest, risk, or decision biases. Instead, full PT accessibility results in prosocial moral judgements and purchasing behaviour. Interestingly, Huang et al. [77] proposed that in VOI reasoning tasks, participants’ responses will tend to be utilitarian, simply because this maximises their odds of a good outcome. However, in Martin et al. [49] utilitarian choices across all behavioural tasks prevailed in the absence of selfishness and uneven odds, indicating that it is perspective-taking accessibility and not risk-averse preferences that results in utilitarian behaviour.

## 4. Conclusions

Philosophers, economists and psychologists have long debated the principles and application of utilitarian theory. Many modern moral psychologists have also examined the circumstances in which people display utilitarian behaviour, with a large focus on how internal processes such as emotional activations and working memory inform participants utilitarian (or non-utilitarian) responses to hypothetical moral dilemmas [12,43,47]. Alternatively, other authors (e.g., [47,48]) consider the importance of enhanced accessibility of utilitarian outcomes through comprehensive information about moral actions and consequences as a predictor of utility maximisation in moral choices.

In this review we have focused on the latter and described recent research which presents participants with novel AV crash scenarios as a new way to measure utilitarian preferences [49,60,77]. These new moral dilemmas involve PT tasks and we have accordingly argued that based on VOI principles [75], care must be taken when presenting scenarios to participants. In particular, Martin et al. [49] demonstrate that presenting participants with the perspective of both a car passenger and a pedestrian (full PT accessibility) during a hypothetical AV collision results in utilitarian preferences across multiple judgement tasks. However, presenting the perspective of only the car passenger (partial PT accessibility) results in the social dilemma exemplified by Bonnefon et al. [60]; people do not want to buy the utilitarian car that they judge to be the most moral [49]. Interestingly, the enhanced and consistent utilitarian behaviour in response to dilemmas with full PT accessibility occurs in the absence of uneven odds and empirically induced self-interest. Therefore, utilitarian strategy is what motivates their utilitarian behaviour, not risk-averse precautionary preferences. Here, we have united two important philosophical concepts—utilitarianism and VOI reasoning. We accordingly argue that in order to measure participants’ prosocial utilitarian behaviour in the absence of bias, perspective-taking accessibility (a new type of VOI with even odds that do no trigger self-interest, risk related preferences or decision biases) is necessary. Accordingly, future research should explore human decision-making that utilises this new type of veil of ignorance, which does not tolerate or induce self-interest and decision biases (e.g., particular risk preferences).
